# Associations between quality indicators of internal medicine residency training programs

**DOI:** 10.1186/1472-6920-11-30

**Published:** 2011-06-08

**Authors:** Stephen D Sisson, Sarah S Casagrande, Deepan Dalal, Hsin-Chieh Yeh

**Affiliations:** 1 Department of Medicine, The Johns Hopkins University School of Medicine, Baltimore, MD, USA; 2 Social and Scientific Systems, Bethesda, MD, USA; 3 Department of Medicine, The Cleveland Clinic, Cleveland, OH, USA

**Keywords:** program quality, Residency Review Committee, American Board of Internal Medicine Certifying Examination

## Abstract

**Background:**

Several residency program characteristics have been suggested as measures of program quality, but associations between these measures are unknown. We set out to determine associations between these potential measures of program quality.

**Methods:**

Survey of internal medicine residency programs that shared an online ambulatory curriculum on hospital type, faculty size, number of trainees, proportion of international medical graduate (IMG) trainees, Internal Medicine In-Training Examination (IM-ITE) scores, three-year American Board of Internal Medicine Certifying Examination (ABIM-CE) first-try pass rates, Residency Review Committee-Internal Medicine (RRC-IM) certification length, program director clinical duties, and use of pharmaceutical funding to support education. Associations assessed using Chi-square, Spearman rank correlation, univariate and multivariable linear regression.

**Results:**

Fifty one of 67 programs responded (response rate 76.1%), including 29 (56.9%) community teaching and 17 (33.3%) university hospitals, with a mean of 68 trainees and 101 faculty. Forty four percent of trainees were IMGs. The average post-graduate year (PGY)-2 IM-ITE raw score was 63.1, which was 66.8 for PGY3s. Average 3-year ABIM-CE pass rate was 95.8%; average RRC-IM certification was 4.3 years. ABIM-CE results, IM-ITE results, and length of RRC-IM certification were strongly associated with each other (p < 0.05). PGY3 IM-ITE scores were higher in programs with more IMGs and in programs that accepted pharmaceutical support (p < 0.05). RRC-IM certification was shorter in programs with higher numbers of IMGs. In multivariable analysis, a higher proportion of IMGs was associated with 1.17 years shorter RRC accreditation.

**Conclusions:**

Associations between quality indicators are complex, but suggest that the presence of IMGs is associated with better performance on standardized tests but decreased duration of RRC-IM certification.

## Background

There is no generally accepted single measure that defines the quality of an internal medicine residency training program [1-3]. "Quality" is generally determined in large part by the perspective from which a residency training program is viewed, be that by the trainees, the trainers, the regulators, or society [2]. ABIM-CE pass rates are commonly used as a measure of program quality, and are used by the RRC-IM as among the criteria for accreditation of residency training programs [4,5].

In addition to ABIM-CE pass rate, there are also other potential candidates for program quality indicators. The Internal Medicine In-Training Exam (IM-ITE), administered by nearly all internal medicine residency training programs, serves as a self-assessment tool for residents and as a program evaluation tool for program directors [6,7]. IM-ITE results have been shown to correlate well with ABIM-CE scores, and may serve as an additional indicator of program quality [4-8]. When residency program directors were surveyed on indicators of program quality, they identified institutional support and stability and completeness of key program faculty as among the most important indicators of program quality [1]. Others have shown that the number of departmental faculty, clinical work required of program directors, and the amount of financial support provided by the pharmaceutical industry correlated with training program ABIM-CE pass rates, and therefore served as additional indicators of program quality [9,10].

The associations and causality between most proposed indicators of program quality are unknown. We surveyed internal medicine residency training programs on specific proposed indicators of program quality with the objective to determine potential associations among them, serving as a basis for future study on causality.

## Methods

### Survey

A survey was developed using indicators of program quality as defined by literature review (Additional file 1) [1-4,8-14]. Variables surveyed included hospital type, number of Department of Medicine faculty, size of training program, proportion of trainees that were international medical graduates (IMGs), IM-ITE scores, three year ABIM-CE first try pass rates, duration of most recent RRC-IM certification, proportion of program director work devoted to clinical duties, use of pharmaceutical funding to support resident education, presence of a primary care track, and proportion of graduates entering a subspecialty fellowship. The survey was distributed via email to 67 program directors at internal medicine residency training programs that had subscribed to the Johns Hopkins Internet Learning Center during the 2006-2007 academic year. The survey was distributed via email and collected by email and fax between January and May 2008, with questions specific to the 2006-2007 academic year. This study was approved by the Johns Hopkins School of Medicine institutional Review Board.

### Statistical analysis

The distribution of program types (e.g., university; community-based hospital) that responded to the mailed questionnaire was compared to the distribution of nationally accredited internal medicine programs. National data were obtained from the American Medical Association's (AMA) Fellowship and Residency Electronic Interactive Database (FREIDA) [15]. Programs that responded to the survey were characterized using descriptive statistics (mean, median, range). The associations between ABIM exam passing rate and the IM-ITE percentile rank score for 2^nd ^and 3^rd ^year residents as well as the number of years of program certification (RRC-IM) were examined by the Spearman rank correlation.

The main dependent variables of interest were IM-ITE percentile scores and years of RRC certification. The independent variables of interest were program characteristics. The distribution of IM-ITE percentile scores and years of RRC certification were approximately normal, and modeled as continuous outcomes. Percentage of IMG, percentage of program director's clinical duty, number of faculty and number of residents were analyzed as both continuous variables and dichotomized variables using median values. To evaluate the associations between program characteristics and quality indicators, univariate linear regressions were used first, to compare the mean IM-ITE percentile score and mean years of RRC certification with each program characteristic. Factors associated with IM-ITE and mean years of program certification with a pre-specified p-value of 0.2 in the univariate analysis were subsequently included in the multivariable models to assess the associations with quality indicators after adjustment for potential covariates.

All tests of significance were two-tailed, with an alpha level of 0.05. Analyses were performed using Stata/SE (College station, TX, Version 10.0).

## Results

### Program characteristics

Of the total 67 surveys sent, 51 internal medicine residency training programs completed and returned the questionnaires (response rate 76.1%). Six programs (9%) declined to participate, and no response was received from 10 programs (14.9%). Of the 51 responding programs, 17 (33.3%) were university hospitals and 34 (67.7%) were other types of hospitals. This distribution of program type is similar to non-participating programs in the nationally accredited internal medicine training programs, of which 106 (32.2%) are university hospitals, and 223 (67.8%) are other types of hospitals (chi-square p = 0.93) [15].

Of the 51 responding programs, nine (17.7%) had a primary care track and 34 (66.7%) had the majority of graduates enter subspecialty fellowship training. Twenty seven programs (52.9%) used funds supplied by pharmaceutical companies to support resident education. On average, 44% of residents were international graduates. The proportion of IMGs was higher in the non-university hospitals than in the university hospitals (52% vs. 27%, p = 0.02). Program directors spent 30% of time in weekly clinical duty and 14% of time on annual clinical activities. The mean IM-ITE raw score for PGY2s was 63.1%, and the mean rank percentile was 61.3. For PGY3s, the mean raw score on the IM-ITE was 66.8%, and the mean rank percentile was 58.8 (Table 1).

**Table 1:** Program characteristics

Program characteristic	Mean	Median	Range
Number of full-time salaried Department of Medicine faculty	101	28	0-500

Number of residents	68	54	16-162

% of residents that are international medical graduates (IMGs)	44	46.5	0-100%

Program director% time spent in weekly clinical activities	30.4	30.0	2-70%

Program director% time spent in annual clinical activities	14.3	12.0	2-50%

Years certification granted last RRC-IM review	4.3	4.0	1-10 years

PGY2 IM-ITE average raw score	63.1	63.0	57.0-73.0

PGY2 IM-ITE average percentile rank	61.3	66.5	12-99

PGY3 IM-ITE average raw score	66.8	67.0	61-78

PGY3 IM-ITE average percentile rank	58.8	63.0	14-99

Passed ABIM-CE first try, past 3 years	95.8	96.0	85-100%

### Associations between IM-ITE rank scores, RRC certification, and ABIM-CE pass rates

A training program with lower IM-ITE rank score among PGY2 or PGY3 housestaff was strongly associated with lower ABIM-CE pass rates, with Spearman correlations of 0.60 (p < 0.0001) and 0.49 (p = 0.002) for PGY2 and PGY3, respectively. A positive association was also shown between shorter RRC-IM certification and lower ABIM-CE pass rates, with correlation of 0.29 (p = 0.04).

### Associations between quality indicators and program characteristics

Mean PGY3 IM-ITE scores relative to selected program characteristics are depicted in Table 2. When mean PGY3 IM-ITE percentile rank scores were compared relative to other program characteristics in univariate analysis, PGY3 percentile rank scores were significantly higher in programs that accepted pharmaceutical funding, in programs that had higher proportions of IMG residents, and in programs at which program directors had lower clinical workloads. This conclusion was not different when the percentage of IMG residents was analyzed as a continuous variable (beta-coefficient = 2.06, p = 0.05, per 10% increase in IMG), with the exception that the association between PGY3 percentile rank score and program directors' clinical workload became non-significant when the workload was analyzed as a continuous variable (beta coefficient = --11.37, p = 0.11, per 20% increase in duty hours). In subsequent multivariable linear regressions, we found that receiving pharmaceutical support remained significantly associated with higher PGY3 IM-ITE rank score, after considering proportion of IMGs and program director's clinical activities.

**Table 2:** Univariate and Multivariable Regression of PGY3 IM-ITE scores, RRC Certification, and other Program Characteristics

	Mean PGY3 IM-ITE Percentile Rank Score				Years of RRC Certification			
	**Univariate Model** **β coefficient (95% CI)**	**P Value**	**Multivariable Model†** **β coefficient (95% CI)**	**P Value**	**Univariate Model** **β coefficient (95% CI)**	**P Value**	**Multivariable Model‡** **β coefficient** **(95% CI)**	**P Value**

**Hospital** **(university vs. non university)**	4.67 (-2.11 to 11.46)	0.17	4.21 (-11.02 to 19.45)	0.18	0.39 (-.71 to 1.50)	0.48		

**Pharma support** **(yes vs. no)**	20.23 (6.49 to 33.98)	0.005	15.26 (1.42 to 29.10)	0.032	-0.55 (-1.57 to 0.47)	0.29		

**Number of faculty** **(more than 28 vs. 28 or less)**	4.11 (-11.02 to 19.24)	0.59			0.97 (-0.02 to 1.96)	0.05	0.17 (-1.02 to 1.36)	0.78

**Number of residents** **(more than 54 vs. 54 or lower)**	1.34 (-13.88 to 16.57)	0.86			1.07 (0.09 to 2.06)	0.03	0.72 (-0.42 to 1.87)	0.21

**IMG** **(more than 46% vs. 46% or less)**	19.42 (5.59 to 33.24)	0.007	13.04 (-1.73 to 27.82)	0.082	-1.38 (-2.34 to -.43)	0.005	-1.17 (-2.18 to -.17)	0.02

**Program directors' workload** **(more than 30% vs. 30% or less)**	-16.53 (-32.34 to -0.73)	0.04	-10.79 (-26.70 to 5.12)	0.18	-.03 (-1.14 to 1.08)	0.96		

† Model's constant has coefficient 50.45, R^2 ^= 0.33

‡ Model's constant has coefficient 4.45, R^2 ^= 0.20

Length of RRC-IM certification relative to selected program characteristics is also shown in Table 2. Programs had significantly longer RRC-IM certification cycles if they had smaller proportions of IMG residents, larger numbers of residents, or larger number of faculty. The results were not different when the percentage of IMG residents and the total number of residents were analyzed as continuous variables (beta coefficient = --0.17, p = 0.017, per 10% increase in IMG; beta-coefficient = 0.17, p = 0.045, per 10 residents increase), whereas the association between RRC-IM certification years and number of faculty became non-significant when faculty size was analyzed as a continuous variable (beta-coefficient = 0.06, p = 0.09, per 10 faculty increase). Program director workload, use of pharmaceutical funding, and program type (University vs. other) was not significantly associated with the length of RRC-IM certification. In subsequent multivariable linear regressions, a higher proportion of IMGs was associated with 1.17 years shorter in RRC (p = 0.02) in the adjusted model that included the number of residents and faculty size. This significance remained when the percentage of IMG residents, the number of faculty, and the number of residents were analyzed as continuous variables. After further adjustment for PGY3 IM-ITE percentile score, the association between high IMG percentage and shorter RRC accreditation length remains significant (p = 0.005).

When ABIM-CE pass rates were compared relative to program characteristics, there were no significant differences in these pass rates based on selected program characteristics. Mean PGY2 IM-ITE rank scores were also compared relative to program characteristics. Mean PGY2 IM-ITE rank scores were higher in programs at which program directors had lower clinical workloads, although this association was not statistically significant (69.7^th ^percentile vs. 56.6^th ^percentile; p = 0.08); no other differences were statistically significant.

Faculty to resident ratios were also calculated for each program, and compared to the other quality indicators. A higher faculty-to-resident ratio (defined as above 0.57, the median) was observed at university hospitals relative to all other hospital types (66.7% vs. 6.7%, p < 0.0001), were associated with lower program director weekly clinical duties (25.5% vs. 35.8% effort, p = 0.0073), and a lower proportion of IMG graduates (30.9% vs. 59.4%, p = 0.005).

### Summary of associations of potential quality indicators and program characteristics

**Figure 1:**
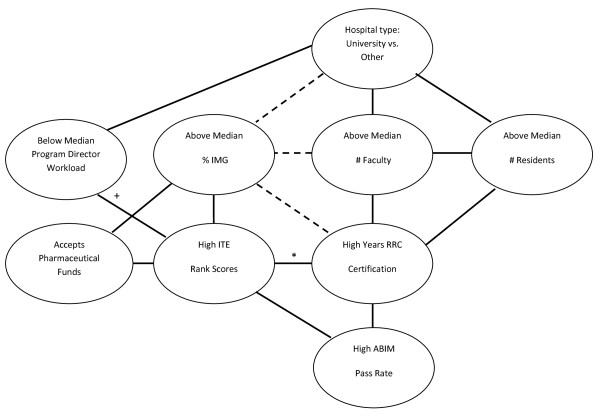
**Summary of associations between program quality indicators**. Solid line: Association (p < 0.05) Dashed line: Inverse association (p < 0.05). *Significant for PGY2 scores but not PGY3 scores +Significant for PGY3 scores but not PGY2 scores

Pair-wise associations among potential program quality indicators and program characteristics are summarized in Figure 1. ABIM-CE pass rates were positively associated with PGY2 and PGY3 IM-ITE percentile rank scores, and with years of RRC-IM certification (all p < 0.05). ABIM-CE pass rates were not associated with other surveyed program quality indicators. High IM-ITE rank scores were associated with programs that accept pharmaceutical funding, had higher proportions of IMG graduates, longer RRC-IM certification cycles (for PGY2 scores only), higher ABIM-CE pass rates, and (for PGY3 IM-ITE scores only), and below median program director clinical workload (all p < 0.05). High proportions of IMG graduates were negatively associated with university hospitals, with an above median number of faculty, and with longer RRC-IM certification (p < 0.05). A high proportion of IMG graduates was associated with programs that accept pharmaceutical funds (p < 0.05). Length of RRC-IM certification was negatively associated with above median proportions of IMG trainees (p < 0.05), and was positively associated with above median numbers of full time faculty and residents (p < 0.05).

## Discussion

In looking for associations between program quality indicators, our results confirm the association between IM-ITE scores, ABIM-CE pass rates, and years of RRC certification, and extends knowledge of associations between other program quality indicators. When looking for associations between ABIM-CE pass rates and other indicators of program quality, there were no significant differences in ABIM-CE pass rates relative to hospital type, proportion of IMG graduates, program director workload, program size, faculty size, or acceptance of pharmaceutical funding. Since the range of ABIM-CE pass rates in our surveyed programs was so narrow, our study may have lacked the power to detect any meaningful associations between ABIM-CE pass rates and other indicators. The range of IM-ITE scores (both raw scores and percentile rank scores) in surveyed programs was wider, and we were able to demonstrate associations between IM-ITE scores and other quality indicators. We found that programs with larger percentages of IMG graduates had higher PGY3 IM-ITE rank scores (69^th ^percentile vs. 49^th ^percentile), consistent with the findings of others [6]. IM-ITE scores were higher in programs that accepted pharmaceutical funding. PGY3 IM-ITE scores were also higher in programs at which program directors had lower clinical workloads.

In our univariate model, we found significant associations between length of RRC certification and markers of program size (i.e. large faculty or number of residents). Programs with higher numbers of residents and programs with higher number of faculty had longer RRC certification than did smaller programs and in those with fewer faculty. We did not, however, find an association between hospital type and length of RRC certification, which contrasts with the findings of others [16]. Larger programs of either type (i.e. university vs. non-university) perhaps had more resources to comply with ACGME requirements. However, these resources are not being used to decrease the clinical workload of program directors; program directors' clinical workload is no less at these larger programs than it is at smaller programs. While some of the demonstrated associations between program characteristics and length of RRC certification are consistent with the most basic goals of residency training (i.e. to train physicians to deliver quality care, as represented by ABIM-CE pass rates), it is unclear why programs with higher numbers of residents and faculty have longer RRC certification.

Among the strongest associations demonstrated was the negative association between a high proportion of IMG graduates and longer RRC certification. This negative association was also present in our multivariable model, in which we corrected for number of residents, number of faculty, and IM-ITE scores. We found that programs with higher proportions of IMGs tend to be non-university hospitals with fewer full-time faculty, and that accept pharmaceutical funding to support training. This may suggest fewer resources to comply with ACGME certification requirements, even though these programs demonstrate successful medical knowledge outcomes among trainees (i.e. IM-ITE scores; ABIM-CE pass rates).

Strengths of our study include survey results from a group of internal medicine training programs whose distribution mirrors that of all internal medicine residency training programs, and with a satisfactory response rate on the survey. Nevertheless, several limitations deserve mention. First, the small variation of ABIM-CE pass rates among surveyed programs limited our ability to detect associations between program quality indicators and ABIM-CE pass rates. In addition, that the average ABIM-CE pass rates and IM-ITE scores in responding programs was greater than 50% suggests that although the distribution of responding programs mirrored that of all programs, responding programs differed in some way from all programs, and results may not be generalizable to all residency programs. Our survey was limited to those potential indicators suggested by the medical literature, and may have missed other program characteristics that are associated with quality, yet were not identified. Our survey also used self-reported ABIM-CE pass rates and IM-ITE performance, which may have been less accurate. Some of the quality indicators studied were aggregate results of individual data (e.g., IM-ITE scores; ABIM pass rates), which is a limitation of this and related studies. Finally, we studied associations, not causality. We do not know whether addressing a program characteristic such as program director workload or faculty size or faculty-to-resident ratios will improve educational outcomes such as IM-ITE scores or ABIM-CE pass rates or length of RRC certification, but our results may form the basis for future study.

## Conclusions

In conclusion, we found that commonly cited indicators of program quality (ABIM-CE pass rates, ACGME certification, and IM-ITE performance) are closely associated, and that the associations between other potential indicators of program quality are complex. Even when correcting for other variables, programs with a high percentage of IMG graduates have a shorter length of RRC certification. Further study is needed to understand the factors underlying the associations observed in this study.

## Abbreviations

ABIM-CE: American Board of Internal Medicine Certifying Examination; ACGME: Accreditation Council for Graduate Medical Education; AMA: American Medical Association; FREIDA: Fellowship and Residency Electronic Interactive Database; IMG: International Medical Graduate; IM-ITE: Internal Medicine In-Training Examination; PGY: Post-Graduate Year; RRC-IM: Residency Review Committee in Internal Medicine

## Competing interests

The authors declare that they have no competing interests.

## Authors' contributions

Data collection was performed by SS and DD. Data interpretation, preparation and critical revision of manuscript and approval of final manuscript were performed by all authors.

## Pre-publication history

The pre-publication history for this paper can be accessed here:

http://www.biomedcentral.com/1472-6920/11/30/prepub
